# Dual Organellar Targeting of Aminoacyl-tRNA Synthetases in Diatoms and Cryptophytes

**DOI:** 10.1093/gbe/evv095

**Published:** 2015-05-20

**Authors:** Gillian H. Gile, Daniel Moog, Claudio H. Slamovits, Uwe-G. Maier, John M. Archibald

**Affiliations:** ^1^Department of Biochemistry and Molecular Biology, Dalhousie University, Halifax, Nova Scotia, Canada; ^2^LOEWE Centre for Synthetic Microbiology (SYNMIKRO), Philipps University Marburg, Germany; ^3^Program in Integrated Microbial Biodiversity, Canadian Institute for Advanced Research, Toronto, Ontario, Canada; ^4^Laboratory for Cell Biology, Philipps University Marburg, Germany; ^5^Present address: Department of Biochemistry and Molecular Biology, Dalhousie University, Halifax, Nova Scotia, Canada

**Keywords:** *Guillardia*, *Phaeodactylum*, pheRS, PPC, protein targeting, *syfB*

## Abstract

The internal compartmentation of eukaryotic cells not only allows separation of biochemical processes but it also creates the requirement for systems that can selectively transport proteins across the membrane boundaries. Although most proteins function in a single subcellular compartment, many are able to enter two or more compartments, a phenomenon known as dual or multiple targeting. The aminoacyl-tRNA synthetases (aaRSs), which catalyze the ligation of tRNAs to their cognate amino acids, are particularly prone to functioning in multiple subcellular compartments. They are essential for translation, so they are required in every compartment where translation takes place. In diatoms, there are three such compartments, the plastid, the mitochondrion, and the cytosol. In cryptophytes, translation also takes place in the periplastid compartment (PPC), which is the reduced cytoplasm of the plastid’s red algal ancestor and which retains a reduced red algal nucleus. We searched the organelle and nuclear genomes of the cryptophyte *Guillardia theta* and the diatoms *Phaeodactylum tricornutum* and *Thalassiosira pseudonana* for aaRS genes and found an insufficient number of genes to provide each compartment with a complete set of aaRSs. We therefore inferred, with support from localization predictions, that many aaRSs are dual targeted. We tested four of the predicted dual targeted aaRSs with green fluorescent protein fusion localizations in *P. tricornutum* and found evidence for dual targeting to the mitochondrion and plastid in *P. tricornutum* and *G. theta*, and indications for dual targeting to the PPC and cytosol in *G. theta.* This is the first report of dual targeting in diatoms or cryptophytes.

## Introduction

Diatoms and cryptophytes are very distantly related algal groups that share the characteristic of having secondary plastids of red algal origin (Bachvaroff et al. 2005; Burki et al. 2012). In both groups the plastids are bound by four membranes and retain a vestigial eukaryotic cytoplasm, called the periplastid compartment (PPC), between the inner and outer pairs of membranes (Gibbs 1981). In diatoms, the PPC is minimal, housing only a handful of proteins, all of which are nucleus-encoded (Moog et al. 2011). In cryptophytes, however, the PPC retains a miniaturized red algal nucleus called a nucleomorph, which encodes roughly 450 PPC-resident proteins and 30 plastid-targeted ones (Douglas et al. 2001; Lane et al. 2007; Tanifuji et al. 2011; Moore et al. 2012). A further estimated 2,400 PPC proteins are encoded in the nucleus and contribute to nucleomorph maintenance and expression as well as starch synthesis in the PPC (Curtis et al. 2012).

Protein targeting to these complex plastids is strikingly similar in diatoms and cryptophytes. Nucleus-encoded proteins destined for the plastid have an amino-terminal signal peptide, which mediates cotranslational transport across the outermost membrane, followed by a transit peptide-like sequence (TPL), which is necessary for transport across the remaining three membranes (Wastl and Maier 2000; Apt 2002). Together, the signal peptide and TPL are often called a bipartite targeting sequence, or BTS. If the TPL begins with an aromatic amino acid, the protein can cross the innermost pair of membranes into the plastid stroma. If not, the protein will be retained in the PPC (Kilian and Kroth 2005; Gould, Sommer, Hadfi, et al. 2006; Gruber et al. 2007). This similarity in targeting peptides between cryptophytes and diatoms likely reflects the relatedness of the import components in these two algal lineages. Transport across the second-to-outermost membrane, also known as the periplastid membrane, is mediated by the symbiont-specific ERAD-like machinery (Sommer et al. 2007; Hempel et al. 2009; Stork et al. 2012). Transport across the inner two membranes most likely occurs through TOC and TIC transporters (translocons of the outer and inner chloroplast membranes, respectively) similar to those found in primary algal plastids, though no TOC-like proteins have yet been identified in cryptophytes (Kalanon and McFadden 2008; Bolte et al. 2009; Bullmann et al. 2010). Proteins destined for mitochondria are also equipped with an N-terminal extension, in this case called a presequence (Schmidt et al. 2010).

Signal peptides, TPLs, and presequences typically lack sequence conservation and are instead differentiated by characteristics such as charge, hydrophobicity, secondary structure, and amino acid composition. In general, signal peptides have a positively charged N-terminus, a hydrophobic central region, and a polar, noncharged C-terminus terminating in the three-residue Von Heijne motif (Von Heijne 1983, 1986; Emanuelsson et al. 2007). These characteristics are found throughout eukaryotes and are fairly reliably predicted by programs such as SignalP (Bendtsen et al. 2004). Likewise, the positive charge and amphipathic alpha-helices of mitochondrial presequences are recognizable in diverse eukaryotes using programs, such as TargetP and Mitoprot (Claros and Vincens 1996; Emanuelsson et al. 2000; Schmidt et al. 2010). Plastid-targeting peptides, however, tend to have lineage-specific characteristics, such as elevated serine and threonine frequencies in the transit peptides of land plants, or an initial aromatic amino acid in those of glaucophytes and red algae and the TPLs of certain algae with red alga-derived complex plastids (Patron and Waller 2007). As a result, transit peptide/TPL prediction is difficult and many lineage-specific prediction programs have been developed in attempt to improve performance, for example, ApicoAP for apicomplexans (Cilingir et al. 2012), PredAlgo for green algae (Tardif et al. 2012), and HECTAR and ASAFind for diatoms and cryptophytes (Gschloessl et al. 2008; Gruber et al. 2015).

Subcellular localization prediction is further complicated by dual targeting, in which the protein products of a single gene can be targeted to and function in multiple subcellular compartments. With over 100 experimentally localized dual-targeted proteins known in plants and an estimated one-third of yeast mitochondrial proteins functioning in additional compartments, it is becoming apparent that dual targeting is an important aspect of cellular protein trafficking (Ben-Menachem et al. 2011; Carrie and Small 2013). Dual-targeted proteins can have diverse functions in any combination of subcellular locations. For example, mitochondria and peroxisomes often share enzymes of the citric acid cycle (Ast et al. 2013), mitochondria and plastids share components of DNA replication and transcription (Carrie and Small 2013), and nuclei and plastids share transcription factors that allow coordinated light-induced gene expression (Krause and Krupinska 2009). Triple and even quadruple-targeted proteins are known (Von Braun et al. 2007; Ralph 2007). Dual targeting can be a matter of life or death: mutation in a dual-targeted protoporphyrinogen oxidase conferred herbicide resistance in the noxious weed *Amaranthus tuberculatus* (Patzoldt et al. 2006). Dual targeting is also widespread, having been discovered not only in yeast and *Arabidopsis*, but also in humans (Tolkunova et al. 2000), in parasites such as trypanosomatids and apicomplexans (Rinehart et al. 2004; Günther et al. 2007; Pino et al. 2007; Ponpuak et al. 2007; Saito et al. 2008; Pham et al. 2014), and even in one lineage of complex algae, the chlorarachniophytes (Hirakawa et al. 2012). To date, no examples of dual targeting are known from diatoms or cryptophytes.

There are a number of ways that dual targeting can be achieved. To generate proteins with different N-terminal targeting information, a gene can have multiple transcription start sites, as in the genes for apicoplast/cytosol aminoacyl-tRNA synthetases (aaRSs) in apicomplexans (Jackson et al. 2012). A gene’s transcript can have multiple spliceoforms, as for the mitochondrion/cytosol human lysRS (Tolkunova et al. 2000). There can be multiple translation start sites, as in transcripts for plastid/mitochondrion hisRS and glyRS in chlorarachniophytes (Hirakawa et al. 2012). Alternatively, a single locus might have only one type of protein product, in which case dual targeting is achieved by an ambiguous targeting peptide that can be recognized by the transporters of multiple subcellular compartments. Most land plant aaRSs are dual targeted to mitochondria and plastids in this latter fashion (Duchêne et al. 2005).

AaRSs feature prominently in studies of dual organelle targeting because they are essential for translation and they are not encoded in most organelle genomes (Chatton et al. 1988; Mudge et al. 1998; Duchêne et al. 2009). This enables a logical framework for inferring cases of dual targeting: If there are insufficient nuclear loci to provide each translationally active compartment with its own complete set of aaRSs, two or more compartments must share. In diatoms, protein translation takes place in three subcellular compartments, the cytosol, plastid, and mitochondrion. Thus for each compartment to use a full complement of 20 amino acids for translation, 60 distinct aaRS proteins should be needed in the absence of dual targeting. Cryptophytes require an additional set for the PPC, bringing their expected total to 80. These expected aaRS totals are approximate, however. Plastids and/or mitochondria sometimes use amidotransferases to modify mischarged glu-tRNA^gln^ and/or asp-tRNA^asn^ to gln-tRNA^gln^ and asn-tRNA^asn^, in which case they do not require glnRS and/or asnRS (Ibba et al. 2005; Frechin et al. 2009). Some organelles, notably the mitochondria of apicomplexans, import charged tRNAs, in which case we expect the tRNAs in question to be missing from the organelle genome (Pino et al. 2010). Here, we have taken advantage of this logical framework to identify potential cases of aaRS dual targeting in cryptophytes and diatoms. We have characterized the N-termini of all the nucleus-encoded aaRSs in the cryptophyte *Guillardia theta* and the diatoms *Thalassiosira pseudonana* and *Phaeodactylum tricornutum* in order to predict which subcellular compartments are sharing the same gene, and we have tested our targeting predictions with homologous and heterologous green fluorescent protein (GFP)-fluorescence localization studies in *P. tricornutum.*

## Materials and Methods

### Gene Finding and Model Assessment

Genomes of the cryptophyte *G. theta* (Curtis et al. 2012) and the diatoms *P. tricornutum* and *T. pseudonana* (Armbrust et al. 2004; Bowler et al. 2008) were searched for aaRSs by tBLASTn through the Joint Genomes Institute (JGI) genomes portal (Grigoriev et al. 2012) using previously characterized aaRS amino acid sequences from eukaryotes, bacteria, and archaea as queries. We also searched all seven organelle genomes (three plastids, three mitochondria, and one nucleomorph) for aaRSs and checked that a full complement of tRNAs was encoded and that a full complement of amino acids would be required to translate expressed organelle genes (Douglas and Penny 1999; Douglas et al. 2001; Oudot-Le Secq et al. 2007; Oudot-Le Secq and Green 2011).

Our search for aaRS genes in the algal nuclear genomes revealed 43 distinct aaRS loci in each diatom and 58 in *G. theta* (table 1). At each locus, there are multiple competing gene models generated by different types of gene finding software (for details, see Curtis et al. 2012, Bowler et al. 2008, and Armbrust et al. 2004). Many of these programs delimit open reading frames (ORFs) that are truncated at the 5′-end, particularly in the case of genes whose products have N-terminal targeting extensions, likely due to of a lack of sequence conservation in these regions (Curtis et al. 2012; Gruber et al. 2015). To overcome this problem, we inspected the genomic sequence upstream of each predicted ORF for in-frame ATG codons. If any were found, we created a new gene model to extend the ORF to the farthest possible upstream ATG without any intervening stop codons. If no upstream, in-frame ATG codons were found, we retained the most complete, computer-generated gene model for further analysis and annotation. Nuclear aaRS gene model information and annotations can be viewed on the JGI genome portal for each organism (http://genome.jgi-psf.org/Guith1/Guith1.home.html for *G. theta*, http://genome.jgi-psf.org/Phatr2/Phatr2.home.html for *P. tricornutum*, or http://genome.jgi-psf.org/Thaps3/Thaps3.home.html for *T. pseudonana*). Gene sequences have also been submitted to GenBank (accession numbers: KP998825–KP998882, KR017885–KR017929, and KR025330–KR025374). (These accessions have been suppressed by GenBank because only the CDS coordinates, and not the sequences themselves, were generated in the current study. All sequences in GenBank format can be found as Supplementary Material online.)

**Table 1:** Localization Predictions and Accession Numbers of Diatom and Cryptophyte aaRSs Examined in This Study

	*Guillardia theta*			*Phaeodactylum tricornutum*			*Thalassiosira pseudonana*		
Protein Name	Gene Name	GenBank Accession	Predicted Localization(s)	Gene Name	GenBank Accession	Predicted Localization(s)	Gene Name	GenBank Accession	Predicted Localization(s)
Alanyl-tRNA synthetase	alaRS1	KP998825	Cytosol	alaRS1	KR017885	Cytosol	alaRS1	KR025330	Cytosol
	alaRS2	KP998826	? (incomplete model)	alaRS2	KR017886	Plastid/mito	alaRS2	KR025331	Plastid/mito
Arginyl-tRNA synthetase	argRS1	KP998827	Cytosol	argRS1	KR017887	Cytosol	argRS1	KR025332	Cytosol
	argRS2	KP998828	Plastid/mito	argRS2	KR017888	Plastid/mito	argRS2	KR025333	Plastid/mito
Asparaginyl-tRNA synthetase	asnRS1	KP998829	Cytosol	asnRS1	KR017889	Cytosol	asnRS1	KR025334	Cytosol
	asnRS2	KP998830	Plastid/mito	asnRS2	KR017890	Plastid/mito	asnRS2	KR025335	Plastid/mito
	asnRS3	KP998831	PPC						
Aspartyl-tRNA synthetase	aspRS1	KP998832	Cytosol	aspRS1	KR017891	Cytosol	aspRS1	KR025336	Cytosol
	aspRS2	KP998833	Mitochondrion	aspRS2	KR017892	Plastid/mito	aspRS2	KR025337	? (incomplete model)
	aspRS3	KP998834	PPC						
Cysteinyl-tRNA synthetase	cysRS1	KP998835	Cytosol	cysRS1	KR017893	Cytosol	cysRS1	KR025338	Cytosol
	cysRS2	KP998836	Plastid/mito or PPC/mito	cysRS2	KR017894	Mitochondrion	cysRS2	KR025339	Mitochondrion
	cysRS3	KP998837	PPC or plastid	cysRS3	KR017895	Plastid	cysRS3	KR025340	Plastid
Glutaminyl-tRNA synthetase	glnRS1	KP998838	Cytosol	glnRS1	KR017896	Cytosol	glnRS1	KR025341	Cytosol
	glnRS2	KP998839	Plastid/mito or PPC/mito	glnRS2	KR017897	Plastid	glnRS2	KR025342	Plastid
	glnRS3	KP998840	PPC or plastid						
Glutamyl-tRNA synthetase	gluRS1	KP998841	Cytosol	gluRS1	KR017898	Cytosol	gluRS1	KR025343	Cytosol
	gluRS2	KP998842	Mitochondrion	gluRS2	KR017899	Plastid/mito	gluRS2	KR025344	Plastid
	gluRS3	KP998843	Plastid						
Glycyl-tRNA synthetase	glyRS1	KP998844	Cytosol	glyRS1	KR017900	Cytosol	glyRS1	KR025345	Cytosol
	glyRS2	KP998845	Mitochondrion/PPC	glyRS2	KR017901	Plastid/mito	glyRS2	KR025346	Plastid/mito
	glyRS3	KP998846	Plastid						
Histidyl-tRNA synthetase	hisRS1	KP998847	Cytosol	hisRS1	KR017902	Cytosol	hisRS1	KR025347	Cytosol
	hisRS2	KP998848	Plastid/mito	hisRS2	KR017903	Plastid/mito	hisRS2	KR025348	Plastid/mito
	hisRS3	KP998849	PPC						
Isoleucyl-tRNA synthetase	ileRS1	KP998850	Cytosol	ileRS1	KR017904	Cytosol	ileRS1	KR025349	Cytosol
	ileRS2	KP998851	Mitochondrion	ileRS2	KR017905	Plastid/mito	ileRS2	KR025350	Plastid/mito
	ileRS3	KP998852	PPC						
Leucyl-tRNA synthetase	leuRS1	KP998853	Cytosol	leuRS1	KR017906	Cytosol	leuRS1	KR025351	Cytosol
	leuRS2	KP998854	Plastid/mito	leuRS2	KR017907	Plastid/mito	leuRS2	KR025352	Plastid/mito
	leuRS3	KP998855	PPC						
Lysyl-tRNA synthetase	lysRS1	KP998856	Cytosol	lysRS1	KR017908	Cytosol	lysRS1	KR025353	Cytosol
	lysRS2	KP998857	Mitochondrion	lysRS2	KR017909	Plastid/mito	lysRS2	KR025354	Plastid/mito
	lysRS3	KP998858	Plastid						
	lysRS4	KP998859	PPC						
Methionyl-tRNA synthetase	metRS1	KP998860	Cytosol	metRS1	KR017910	Cytosol	metRS1	KR025355	Cytosol
	metRS2	KP998861	Plastid/mito	metRS2	KR017911	Plastid/mito	metRS2	KR025356	Plastid/mito
	metRS3	KP998862	PPC						
Phenylalanyl-tRNA synthetase	pheRS1a	KP998863	Cytosol	pheRS1a	KR017912	Cytosol	pheRS1a	KR025357	Cytosol
	pheRS1b	KP998864	Cytosol	pheRS1b	KR017913	Cytosol	pheRS1b	KR025358	Cytosol
	pheRS2	KP998865	Plastid/mito	pheRS2	KR017914	Mitochondrion	pheRS2	KR025359	Mitochondrion
	pheRS3	KP998866	PPC	pheRS3	KR017915	Plastid	pheRS3	KR025360	Plastid
Prolyl-tRNA synthetase	proRS1	KP998867	Cytosol	proRS1	KR017916	Cytosol	proRS1	KR025361	Cytosol
	proRS2	KP998868	Plastid/mito	proRS2	KR017917	Plastid/mito	proRS2	KR025362	Plastid/mito
	proRS3	KP998869	PPC						
Seryl-tRNA synthetase	serRS1	KP998870	Cytosol	serRS1	KR017918	Cytosol	serRS1	KR025363	Cytosol
	serRS2	KP998871	Plastid/mito	serRS2	KR017919	Plastid/mito	serRS2	KR025364	Plastid/mito
Threonyl-tRNA synthetase	thrRS1	KP998872	Cytosol	thrRS1	KR017920	Cytosol	thrRS1	KR025365	Cytosol
	thrRS2	KP998873	Plastid/mito	thrRS2	KR017921	Plastid/mito	thrRS2	KR025366	Plastid/mito
	thrRS3	KP998874	PPC						
Tryptophanyl-tRNA synthetase	trpRS1	KP998875	Cytosol	trpRS1	KR017922	Cytosol	trpRS1	KR025367	Cytosol
	trpRS2	KP998876	Plastid/mito	trpRS2	KR017923	Plastid/mito	trpRS2	KR025368	Plastid/mito
	trpRS3	KP998877	PPC						
Tyrosyl-tRNA synthetase	tyrRS1	KP998878	Cytosol/PPC	tyrRS1	KR017924	Cytosol	tyrRS1	KR025369	Cytosol
	tyrRS2	KP998879	Plastid/mito	tyrRS2	KR017925	Plastid/mito	tyrRS2	KR025370	Plastid/mito
Valyl-tRNA synthetase	valRS1	KP998880	Cytosol	valRS1	KR017926	Cytosol	valRS1	KR025371	Cytosol
	valRS2	KP998881	? (incomplete model)	valRS2	KR017927	Plastid/mito	valRS2	KR025372	Plastid/mito
	valRS3	KP998882	PPC						
Glutamyl-tRNA amidotransferase subunit A				gatA	KR017928	Mitochondrion	gatA	KR025373	Mitochondrion
>Glutamyl-tRNA amidotransferase subunit B				gatB	KR017929	Mitochondrion	gatB	KR025374	Mitochondrion

To assess support for our chosen gene models, we checked whether the 5′-end of the gene model is transcribed by blasting against RNAseq contigs from the Marine Microbial Eukaryote Transcriptome Sequencing Project for *G. theta* (Keeling et al. 2014) and the Diatom expressed sequence tag (EST) database for *P. tricornutum* and *T. pseudonana* (Maheswari et al. 2005, 2009). Most gene models’ 5′-ends were supported by transcript data in *G. theta* (51/58) and *P. tricornutum* (39/43), but the *T. pseudonana* transcript data are sparse and only support the 5′-ends of 11/43 gene models (supplementary tables S1–S3, Supplementary Material online).

### Localization Prediction

Without a detailed map of transcription start sites, we do not know which ATG codon represents the true start of the ORF, or whether multiple ATG codons might serve as start codons thanks to alternate transcription and/or translation initiation sites. We therefore performed localization predictions on all potential N-termini (beginning with each successive methionine residue) before the start of the conserved aaRS domain (supplementary tables S1–S3, Supplementary Material online). AaRS domain boundaries were determined by BLAST alignments to the National Center for Biotechnology Information (NCBI) conserved domain database (Marchler-Bauer et al. 2013). Targeting predictions were made using SignalP 3.0 (Bendtsen et al. 2004), SignalP 4.1 (Petersen et al. 2011), ASAFind (Gruber et al. 2015), TargetP 1.1 (Emanuelsson et al. 2007), Predotar (Small et al. 2004), iPSORT (Bannai et al. 2002), WoLF PSORT (Horton et al. 2007), and Mitoprot (Claros and Vincens 1996).

Localization predictions typically differ according to the program used, and most programs do not account for dual or multiple targeting. In this study, dual targeting was anticipated for one or more members of a given aaRS type unless there were sufficient numbers of genes to provide unique proteins for each subcellular compartment. Diatoms are expected to require three loci to encode distinct copies of each aaRS (one each for the cytosol, mitochondrion, and plastid) whereas *G. theta* is expected to require four (one each for the cytosol, mitochondrion, plastid stroma, and PPC). To infer dual targeting and to predict which subcellular destinations were shared, we first determined the single most likely subcellular destination for each protein based on information from multiple prediction programs. Next, we sought the best candidate protein to supply the missing subcellular destination from each aaRS set by looking for possible ambiguity in targeting peptide characteristics (i.e., disagreement among the prediction programs) and by predicting the destinations of proteins beginning with alternate (i.e., downstream) translation start sites (supplementary tables S1–S3, Supplementary Material online).

### Phylogenetic Analyses

Protein sequences of phenylalanyl-tRNA synthetase (pheRS) alpha and beta subunits (encoded by the genes pheS and pheT) were sought in the whole genomes of 13 prokaryote species chosen to represent the phylogenetic diversity of prokaryotes. Eukaryotic pheRS sequences were sought by BLAST from various databases: *Arabidopsis thaliana* from The Arabidopsis Information Resource (www.arabidopsis.org); *Plasmodium falciparum* from The Eukaryotic Pathogen Database (eupathdb.org); diatoms, *Bigelowiella natans*, and *G. theta* from the JGI web portal (genome.jgi-psf.org); and all other sequences from the NCBI protein database (www.ncbi.nlm.nih.gov/protein). Sequences were aligned with MAFFT (Katoh et al. 2002) and trimmed by eye using SeaView 4.5.3 (Gouy et al. 2010). Maximum-likelihood phylogenetic trees were estimated using RAxML 7.2.5 (Stamatakis 2006) using the LG substitution matrix (Le and Gascuel 2008) with four categories of gamma-approximated rates and empirical amino acid frequencies, and with support estimated from 1,000 bootstrap replicates. A Bayesian phylogeny for each subunit was estimated using PhyloBayes 3.2 (Lartillot and Philippe 2004) from two independent runs of 850,000 generations. Every tenth tree was saved and the first 10,000 trees from each run were discarded as burn-in before the remaining 75,000 trees from each run were used to compute the consensus tree and posterior probabilities. The maximum discrepancy across bipartitions (maxdiff value) observed for the pheRS alpha subunit was 0.024, and for the pheRS beta subunit it was 0.012.

### Sequence Amplification and Cloning

Targeting peptide-coding regions of *P. tricornutum* (Pt) and *G. theta* (Gt) aaRSs were amplified with specific oligonucleotides generating a 5′ *Eco*RI and a 3′ *Bam*HI or *Bgl*II restriction site using standard polymerase chain reaction conditions. Although the targeting sequence encoding region for Pt_ArgRS2 (Phatr2 protein ID 36013, GenBank accession number KR017888) was amplified from gDNA (because this region is already known from EST data to be expressed), those for Pt_AsnRS2 (Phatr2 protein ID 42274, GenBank accession number KR017890) as well as Gt_TyrRS1 (Guith1 protein ID 94450, GenBank accession number KP998878) and Gt_TyrRS2 (Guith1 protein ID 199645, GenBank accession number KP998879) were amplified from cDNA, to confirm expression of these regions and to avoid introns. Primer sequences were as follows. Pt_ArgRS2-F: 5′-GAA TTC ATG TTC CGT TCC TCG GCA ACC G-3′, Pt_ArgRS2-R: 5′-GGA TCC GGC ATC CTG TTT TGC AAA GGG-3′; Pt_AsnRS2-F: 5′-GAA TTC ATG TCG AGA TTC CTG GGT GTG C-3′, Pt_AsnRS2-R: 5′-AGA TCT GGA AAC AGG GCC ATC CAT AGG C-3′; Gt_TyrRS1-F: 5′- GAA TTC ATG TTG TCT GAG CGA ACG AG-3′, Gt_TyrRS1-R: 5′- GAA GAT CTA TCC TCC AGA GCG TTC ATG-3′; Gt_TyrRS2-F: 5′-GAA TTC ATG CTG CGA GGA ACG CTG-3′, Gt_TyrRS2-R: 5′-GAA GAT CTC TTT GAC TTC CCG TCG AAG C-3′ (restriction sites underlined). The amplified leader sequences were cloned by restriction and subsequent ligation in front of *egfp* (enhanced GFP; *Bam*HI/*Hin*dIII) into the multiple cloning site of pPha-NR (GenBank JN180663; *Eco*RI/*Hin*dIII), which allows induced expression of the constructs under control of an endogenous nitrate reductase promoter. Generated constructs were analyzed for correctness through standard Sanger sequencing before they were transformed into the diatom.

### Transformation, Cell Culture, and Induced Protein Expression

Genetic transformation of *P. tricornutum* was performed as described previously (Apt et al. 1996; Zaslavskaia et al. 2000). Transformed cells were cultured under continuous light (80 µmol photons × m^−2^ × s^−1^) at 22 °C on solid (1.3 % agar) f/2 medium containing 1.5 mM NH_4_^+^ as the sole nitrogen source (noninduced conditions). Expression of the leader sequence-GFP fusion proteins was induced by growing positive clones for 48 h on solid f/2 medium containing 0.9 mM NO_3_^−^.

### MitoTracker Staining

Staining of mitochondria was performed using MitoTracker Orange CMTMRos from Molecular Probes (Invitrogen). Cells were pelleted by centrifugation (1.500 × g, 5 min, RT), washed once with PBS (137 mM NaCl, 10 mM Na_2_HPO_4_, 2.7 mM KCl, 1.8 mM KH_2_PO_4_, pH adjusted to 7.4 with HCl), and incubated with 500 mM MitoTracker Orange CMTMRos in phosphate buffered saline at room temperature for 30–45 min in the dark. Cells were washed with PBS twice more, including an incubation of 15 min in the dark during the second wash step. Finally, the cells were resuspended in PBS and analyzed under the confocal microscope.

### Confocal Microscopy

Positive clones were analyzed with a Leica TCS SP2 confocal laser scanning microscope (CLSM) as described previously (Moog et al. 2011). GFP and plastid autofluorescence (PAF) were excited with a 65-mW Ar-laser at 488 nm. Emission was detected between 500 and 520 nm (GFP) and 625 and 720 nm (PAF). The MitoTracker Orange CMTMRos was excited using a 1.2-mW HeNe-laser at a wavelength of 543 nm, whereas emission was detected between 560 and 590 nm.

## Results

### Gene Finding

Our BLAST-based search of the nuclear genomes of two diatoms, *T. pseudonana* (Armbrust et al. 2004) and *P. tricornutum* (Bowler et al. 2008), and the cryptophyte *G. theta* (Curtis et al. 2012) identified 43 aaRS loci in each diatom and 58 aaRS loci in *G. theta.* Although this is substantially fewer than the number required to provide each translationally active compartment with its own unique protein (roughly 60 for each diatom and 80 for the cryptophyte), aaRSs are highly conserved and it is unlikely that any were missed by our search. In *Pl. falciparum*, a search using hidden Markov models failed to uncover any aaRSs not already identified by BLAST (Bhatt et al. 2009).

We also searched all seven organelle genomes of these three species (three mitochondria, three plastids, and one nucleomorph) for aaRSs, because our inference of dual targeting rests on the assumption that an organelle both needs and lacks a given aaRS. The nucleomorph of *G. theta* encodes serRS and the plastid of *P. tricornutum* encodes the pheRS β subunit (Douglas et al. 2001; Oudot-Le Secq et al. 2007). All other aaRSs are missing from the organelle genomes. We next confirmed that codons for all 20 amino acids were used in expressed genes from each organelle genome (data not shown). Finally, we checked for tRNA genes in each organelle. If a tRNA gene is missing from an organelle genome, the possibility remains open that the organelle imports that tRNA in its aminoacylated form and therefore has no need of the corresponding aaRS, as has been demonstrated for *Toxplasma gondii* and inferred for chlorarachniophytes (Pino et al. 2010; Hirakawa et al. 2012). All of the plastid genomes have a full set of tRNA genes (Douglas and Penny 1999; Oudot-Le Secq et al. 2007), as do the diatom mitochondria (Oudot-Le Secq and Green 2011), but the mitochondrial genome of *G. theta* is missing tRNA^lys^ (Curtis et al. 2012) and the nucleomorph is missing tRNA^glu^ (Douglas et al. 2001).

### Diatom aaRSs

The two diatoms show perfect congruence in the number of nuclear genes for each aaRS type. Most aaRSs are encoded by only two distinct loci and are therefore expected to require dual targeting to service all three subcellular compartments in which translation takes place. One exception is cysRS, which has three loci that encode proteins predicted to be targeted to the cytosol, mitochondrion, and plastid, presumably without dual targeting (table 1 and supplementary tables S1–S3, Supplementary Material online). Similarly, glnRS appears not to require dual targeting: Though glnRS is only encoded at two loci in each diatom nuclear genome, one predicted to be cytosolic and one predicted to target the plastid, the presence of two subunits of the glu-tRNA^gln^ amidotransferase with predicted mitochondrial presequences suggests that diatom mitochondria do not need glnRS. This is a common scenario, known from yeast, plants, and humans, though not, for example, in trypanosomatids (Frechin et al. 2009; Araiso et al. 2014), and not in *G. theta* (see below).

**Fig. 1.—:**
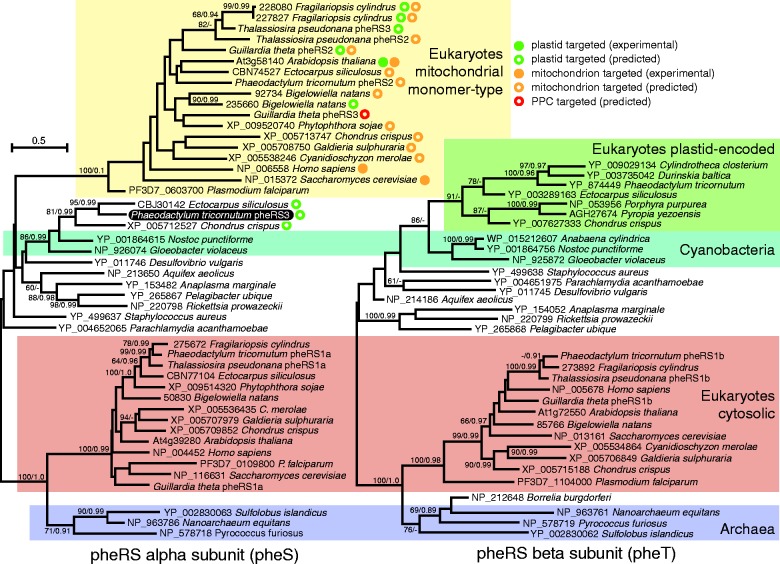
Maximum-likelihood (ML) phylogenies of pheRSs. Left: pheRS alpha subunit, including monomeric type typically found in mitochondria. Right: pheRS beta subunit. Eukaryotes typically encode multiple pheRSs: An alpha and a beta subunit for the cytosol (salmon-colored box, left and right tree, respectively), a monomer-type for the mitochondrion (yellow box), and, in algae, an additional pheRS for the plastid. In the diatoms *T. pseudonana* and *F. cylindrus*, the plastid is served by mitochondrial-type monomeric pheRSs, whereas in *P. tricornutum*, the beta subunit (green box, right tree) is encoded in the plastid genome while the alpha subunit (indicated by white text on black background, left tree) is related to cyanobacterial homologs and therefore appears to be a product of endosymbiotic gene transfer. Numbers at nodes indicate ML bootstrap support out of 1,000 replicates/Bayesian posterior probabilities.

Finally, diatom pheRS does not appear to require dual targeting, but because it can exist either as a monomer or as a heterotetramer of alpha and beta subunits, the required number of loci is variable (Sanni et al. 1991). Each diatom has a total of four pheRS genes, encoding one eukaryote-type alpha and one eukaryote-type beta subunit each predicted to be cytosolic, a monomer-type pheRS predicted to be mitochondrial, and an additional potentially plastid-targeted pheRS. In *T. pseudonana*, the predicted plastid-targeted pheRS (pheRS3, Thaps3 protein ID 24163, GenBank accession number KR025360) is also of the mitochondrial monomer type, whereas in *P. tricornutum* (pheRS3, Phatr2 protein ID 56835, GenBank accession number KR017915), it is a prokaryote-type alpha subunit; the prokaryote-type beta subunit is encoded in the plastid genome (Oudot-Le Secq et al. 2007). Interestingly, in *T. pseudonana* the plastid-targeted monomer pheRS is derived from a recent duplication of the mitochondrial pheRS gene. This same genetic process has also given rise to plastid-targeted, mitochondrial type pheRSs independently in the diatom *Fragilariopsis cylindrus* and the chlorarachniophyte *B. natans* (fig. 1). The plastid-targeted alpha subunit of *P. tricornutum*, by contrast, branches with cyanobacteria just like the beta subunit still encoded in its plastid genome, and therefore appears to be derived from an endosymbiotic gene transfer (fig. 1).

In the remaining 17 diatom aaRSs, encoded by two loci each, a pattern emerges. One copy lacks an N-terminal extension and/or is predicted to be cytosolic, and the other copy has a predicted signal peptide immediately followed by an aromatic amino acid, as is typical of diatom plastid BTSs. These signal-bearing, putatively plastid-targeted aaRSs were additionally predicted to be mitochondrion-targeted, in one of two ways. Either the signal peptide itself was predicted by other methods to be a mitochondrial presequence, or a truncated version of the protein, starting at the next downstream M residue, was predicted to be mitochondrion-targeted (supplementary tables S1 and S2, Supplementary Material online). In many cases both were true, but the truncated protein had higher mitochondrial targeting scores than the full-length signal peptide. Based on this information, we expect that for the majority of aaRSs in diatoms, one of the two copies is dual targeted to the mitochondrion and plastid.

**Fig. 2.—:**
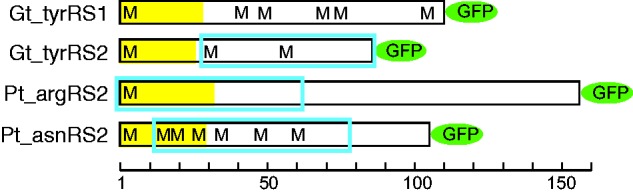
Schematic representation of N-terminal extensions of aaRSs from *G. theta* and *P. tricornutum* chosen for GFP localization experiments, see text for details. Predicted signal peptides are represented by yellow fill, predicted mitochondrial presequences are represented by blue boxes, and the locations of all methionine residues are indicated by M. Bottom axis measures amino acid residues. Green ellipses labeled “GFP” indicate the location of GFP, chosen to correspond to the predicted start of the mature aaRS domain, and are not to scale.

**Fig. 3.—:**
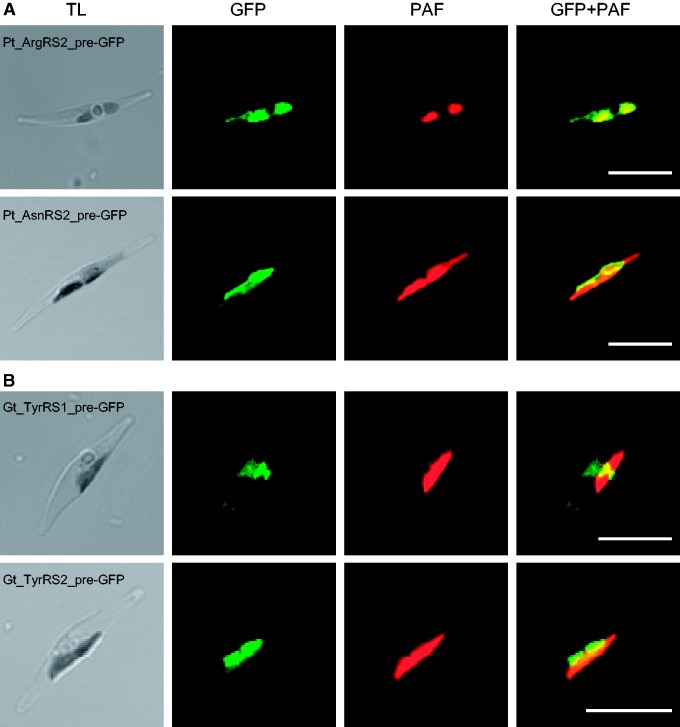
Fluorescence localization of aaRSs. Constructs of N-terminal targeting sequences from aaRSs fused to eGFP were expressed in the diatom *P. tricornutum.* (*A*) Localization of aaRS proteins from *P. tricornutum*, arginyl-tRNA synthetase 2 (Pt_argRS2) and asparaginyl-tRNA synthetase 2 (Pt_asnRS2). GFP fluorescence can be seen in a region adjacent to the plastid as well as colocalizing with PAF, indicative of dual targeting to the mitochondrion and the plastid. (*B*) Localization of tyrosyl-tRNA synthetase proteins from the cryptophyte *G. theta* (Gt_tyrRS1 and Gt_tyrRS2). The Gt_tyrRS1 construct shows diffuse fluorescence throughout the cytosolic area as well as in a small concentrated region characteristic of PPC localization (the “blob-like structure”), seen here as a small, hourglass-shaped, bright green spot. The Gt_tyrRS2 construct shows GFP fluorescence adjacent to and colocalizing with PAF, indicative of dual targeting to the mitochondrion and plastid. TL, transmitted light; Scale bars: 10 μm.

To test this prediction, we chose two *P. tricornutum* putatively dual-targeted aaRSs for localization experiments: asnRS2 (Phatr2 protein ID 42274, GenBank accession number KR017890) because it had the most strongly predicted signal peptide from the first M and the most strongly predicted mitochondrial presequence from the next M, and argRS2 (Phatr2 protein ID 36013, GenBank accession number KR017888) because it was the only signal-bearing aaRS without a downstream M residue before the conserved aaRS domain (fig. 2 and supplementary table S1, Supplementary Material online). We transformed *P. tricornutum* cells with vectors encoding the asnRS2 and argRS2 N-terminal extensions fused to eGFP. After expression of the fusion constructs, in both cases we were able to detect fluorescence not only overlying the autofluorescence of the plastid but also clearly extended alongside the plastid, a pattern that is strongly indicative of dual targeting to the plastid and mitochondrion (fig. 3A). Because the intensity of GFP fluorescence in the plastid was rather faint in comparison to the mitochondrion-localized GFP signal, and because it is known that mitochondria are usually located in close proximity to the plastid in *P. tricornutum* (Prihoda et al. 2012), we additionally stained the positive clones with mitotracker. We observed a clear colocalization of mitotracker with GFP fluorescence, as well as colocalization of GFP with PAF where mitotracker was absent (supplementary fig. S1, Supplementary Material online). However, it should be noted that the plastid-localized GFP fluorescence was not seen in all clones or in all cells of a clone, in which case the localization appeared to be mitochondrial only.

### Cryptophyte aaRSs

Most aaRS types in *G. theta* were represented by three loci (15/20), so we would expect that one of the three loci encodes a dual-targeted protein. Indeed, most of these three-copy aaRSs (9/15) were predicted to have a cytosolic copy, a PPC-targeted copy, and a dual plastid/mitochondrion-targeted copy (supplementary table S3, Supplementary Material online). Nevertheless, one (gluRS) has strongly predicted cytosolic, mitochondrial, and plastid localizations for its three copies, with no suggestion of dual targeting. This may be related to the lack of tRNA^glu^ in the nucleomorph: The PPC might import charged glu-tRNA^glu^ rather than gluRS, as was inferred for glyRS in chlorarachniophytes (Hirakawa et al. 2012). Another (glyRS) has a predicted cytosolic, plastid, and dual PPC/mitochondrial localizations. For the rest of the three-copy aaRSs (4/15), we were unable to predict their localizations with confidence. In one case (valRS), a gene model was incomplete. In two cases (cysRS and glnRS), two of the copies were predicted to carry signal peptides, but because neither copy encoded an aromatic amino acid near the signal cleavage site, we were unable to determine with confidence which copy might be targeted to the plastid. Finally, the predictions for ileRS were weak and conflicting for two of the three copies (supplementary table S3, Supplementary Material online).

What about the five aaRSs not represented by three loci each? LysRS has four loci and the targeting predictions are consistent with one copy for each compartment. The remaining four aaRSs, that is, alaRS, argRS, serRS, and tyrRS, only have two loci each, so we would expect both copies to be dual targeted or else one copy to be triple targeted. In fact, we find that serRS is encoded in the nucleomorph, and the two nucleus-encoded copies are predicted to be cytosolic and dual mitochondrion/plastid targeted. argRS shows no sign of PPC-targeting, though there is no obvious reason why the PPC would not require this aaRS. For alaRS, both copies inexplicably appear cytosolic, as no obvious N-terminal extensions are present. Finally, tyrRS has two signal peptide-bearing copies: One is predicted to be dual plastid/mitochondrion-targeted and the other is predicted to target the PPC. Either copy could function in the cytosol if translated from a downstream ATG.

In order to test our dual plastid/mitochondrion targeting prediction for tyrRS in *G. theta*, and in hopes of determining which of the two copies might additionally function in the cytosol, we tested the localization of eGFP fused to the tyrRS1 and tyrRS2 N-terminal extensions through heterologous expression in *P. tricornutum* (fig. 3*B*). For tyrRS2, which is the predicted plastid/mitochondrion-targeted copy (Guith1 protein ID 199645, GenBank accession number KP998879), we observed fluorescence in both the plastid and the mitochondrion, strongly suggesting dual targeting of this protein. However, as with the native *P. tricornutum* aaRS localizations, the plastid-localized fluorescence (i.e., the GFP fluorescence colocalizing with the PAF) was rather faint compared with the mitochondrial GFP fluorescence, and was not seen in all clones derived from a transformation experiment, nor in all cells of a clone. Similar to the *P. tricornutum* native aaRS-GFP localizations, additional mitotracker staining supported the observed dual mitochondrion/plastid GFP localization of the heterologously expressed Gt_tyrRS2_pre-GFP (supplementary fig. S1, Supplementary Material online).

For *G. theta* tyrRS1, the predicted PPC-targeted copy (Guith1 protein ID 94450, GenBank accession number KP998878), we also observed fluorescence patterns consistent with dual targeting after heterologous expression of its N-terminal extension with GFP in *P. tricornutum.* As shown in figure 3*B *the classical “blob-like structure” typical of PPC localization could be detected. Specifically, the “blob-like structure” is a GFP fluorescence pattern precisely located between the plastid lobes of *P. tricornutum* that does not overlay with the PAF. We also observed diffuse, heterogeneous GFP fluorescence throughout the cytoplasm in the majority of positive clones. We interpret these observations as evidence for dual PPC/cytosol targeting (fig. 3 and supplementary fig. S1, Supplementary Material online). However, we cannot rule out the possibility that the diffuse fluorescence is an artifactual ER localization in this heterologous system, as a similar localization pattern was observed for a predicted PPC-specific protein from the haptophyte *Emiliania huxleyi* when localized heterologously in *P. tricornutum* (Felsner et al. 2011). We also stained several Gt_tyrRS2_pre-GFP positive clones with mitotracker, and did not detect any GFP fluorescence colocalizing with mitochondria (supplementary fig. S1, Supplementary Material online).

## Discussion

In this study, we observed dual plastid/mitochondrion localization of GFP fused to the N-terminal extensions of asnRS2 and argRS2 from *P. tricornutum* and tyrRS2 from *G. theta* and homologously or heterologously expressed in *P. tricornutum* (fig. 3 and supplementary fig. S1, Supplementary Material online). However, this result is complicated by the fact that many cells showed fluorescence in the mitochondrion only. A similar result has been observed in plants: Predicted dual-targeting aaRS transit peptides fused to GFP or RFP showed strong fluorescence in mitochondria but variable and weak fluorescence in plastids, despite unequivocal import of the same fusion proteins into isolated plastids and mitochondria (Duchêne et al. 2005). Why this should happen remains an open question. One contributing factor may be the absence of the mature aaRS domain in the GFP fusion construct. In most cases the N-terminal dual-targeting peptide is sufficient to demonstrate dual targeting of GFP (e.g., Peeters et al. 2000; Berglund et al. 2009; Hirakawa et al. 2012; Jackson et al. 2012), but there are reports of differences in experimental localization results depending on whether or not the mature protein is included. For example, the BTS of an apicomplexan superoxide dismutase (SOD) targets GFP to the mitochondrion, but when the mature protein is included in the GFP fusion construct, dual apicoplast/mitochondrion localization can be observed (Pino et al. 2007). Conversely, the targeting peptide of a plant tRNA nucleotidyltransferase consistently dual-localizes GFP to the plastid and the mitochondrion, but when the mature protein is included, the plastid GFP signal becomes variable and weak (Von Braun et al. 2007). Various other factors have been shown to affect the results of localization experiments for dual-targeted proteins, including choice of vector used for the GFP construct and its expression level (Christensen et al. 2005) and the choice of a homologous or heterologous system for transformation (Fuss et al. 2013; Xu et al. 2013). Given these known difficulties, it seems reasonable to interpret the faint and variable plastid GFP fluorescence observed herein as evidence of dual targeting. Dismissing these results would require an explanation of how translation can occur in the plastid in the absence of aaRSs.

For most of the predicted dual-targeted diatom aaRSs, the N-terminal signal peptide was also weakly predicted to be a mitochondrial presequence, but the mitochondrial prediction was typically stronger after the second methionine residue. This is suggestive of dual targeting by alternate transcription or translation initiation, in which one protein isoform carries a mitochondrial presequence and the other has a signal peptide and TPL for plastid targeting. Dual targeting by this mechanism is known in chlorarachniophytes, where hisRS and glyRS target both mitochondria and complex plastids by alternate translation initiation (Hirakawa et al. 2012). However, one of the predicted dual-targeted aaRSs from *P. tricornutum*, argRS2, lacks a second methionine before the start of the mature protein. This is suggestive of dual targeting through an ambiguous targeting peptide, which must be recognizable by both the signal recognition particle and the mitochondrial import machinery. Precedent for this targeting mechanism also exists: In apicomplexan parasites, an SOD is dual-targeted to the mitochondrion and complex plastid through an ambiguous targeting peptide (Pino et al. 2007). Moreover, an aconitase in the apicomplexan *Toxoplasma gondii* was found to be dual targeted by ambiguity despite having a second methionine residue initiating a more strongly predicted mitochondrial presequence (Pino et al. 2007). This also highlights the difficulty of distinguishing between these two mechanisms. We observed dual targeting of both Pt_argRS2_pre- GFP and Pt_asnRS2_pre-GFP (which has the most strongly predicted signal peptide and the most strongly predicted mitochondrial presequence from the second methionine; supplementary table S1, Supplementary Material online), which leaves open the possibility that both ambiguity and the generation of N-terminal isoforms by alternate transcription or translation contribute to dual targeting in diatoms.

Each aaRS type was represented by the same number of nuclear genes in both of the diatoms. The 43 aaRS genes in each diatom also appear to share the same evolutionary history, that is, the aaRS genes of one diatom branch sister to the corresponding aaRS genes of the other diatom, with one exception. The plastid-targeted pheRS of *T. pseudonana* is derived from a duplication of the mitochondrion-targeted monomeric-type pheRS, whereas the plastid-targeted pheRS of *P. tricornutum* is a cyanobacterial-type alpha subunit that appears to be endosymbiotically derived (fig. 1). The plastid pheRS beta subunit, encoded by the gene *syfB*, has a complicated history of endosymbiotic gene transfer (Ruck et al. 2014). It is found sporadically in red algal and red alga-derived plastid genomes, and therefore appears to be ancestrally plastid-encoded (Reith and Munholland 1995; Hagopian et al. 2004; DePriest et al. 2013; Campbell et al. 2014; Hughey et al. 2014; Tajima et al. 2014). Half of the 22 completed diatom plastid genomes have *syfB.* The rest have lost it, approximately six times independently (Kowallik et al. 1995; Oudot-Le Secq et al. 2007; Lommer et al. 2010; Galachyants et al. 2011; Tanaka et al. 2011; Brembu et al. 2014; Ruck et al. 2014; Sabir et al. 2014). Two of those losses, in *T. pseudonana* and *F. cylindrus*, appear to have been enabled by the targeting of a mitochondrial monomer-type pheRS into the plastid (fig. 1). Interestingly, the *F. cylindrus* mitochondrial pheRS gene appears to be very recently duplicated. Furthermore, the *F. cylindrus* monomer-type pheRSs retain high sequence similarity in the N-terminal regions and are both predicted to be dual targeted to plastids and mitochondria, suggesting that at least in this case, the gain of dual targeting capability preceded the gene duplication. Perhaps this same sequence of events, followed by divergence in targeting peptide specificity, led to the two uniquely targeted monomer-type pheRSs in *T. pseudonana.* In *G. theta*, not only does the plastid use a mitochondrial monomer-type pheRS by dual targeting, but also an additional monomer-type pheRS is predicted to target the PPC (fig. 1).

Whenever dual targeting was predicted for diatom aaRSs, the two compartments targeted were plastids and mitochondria. Such a consistent preference for these two compartments is somewhat surprising, given that dual targeting to plastids and the cytosol is known from apicomplexans (Jackson et al. 2012; Pham et al. 2014), and dual targeting of mitochondria and the cytosol is known from fungi and animals (Chatton et al. 1988; Mudge et al. 1998; Tolkunova et al. 2000; Turner et al. 2000; Rinehart et al. 2004; Tang et al. 2004). In *Arabidopsis*, some aaRSs are dual targeted to plastids and mitochondria whereas others are dual targeted to the cytosol and mitochondria (Duchêne et al. 2005). Perhaps even more surprising is the maintenance of dual targeting in each of these aaRSs for over 100 Myr, since before the divergence of Thalassiosirales and pennate diatoms (Sorhannus 2007), which can be inferred from the phylogenies of the individual aaRSs (supplementary table S4, Supplementary Material online). Gene duplications are known to reinstate unique targeting (Chang et al. 2012), and small changes in targeting peptide sequence can easily alter the target destination (Brydges and Carruthers 2003; Tonkin et al. 2006; Xu et al. 2013), yet diatoms seem to consistently dual target plastids and mitochondria in 17 of 20 aaRSs.

In the cryptophyte *G. theta*, the PPC adds three more possible combinations of compartments for dual targeting, in addition to the possibility of triple targeting, which significantly complicates targeting prediction. Although in diatoms any aaRS with a predicted signal peptide would be expected to target the plastid (diatoms do not possess a genetically active PPC), in *G. theta* a signal peptide can direct a protein to the plastid or to the PPC, depending on the nature of the TPL. Distinguishing between PPC and plastid targeting should be relatively straightforward, given that the discriminating factor is known to be an aromatic amino acid or leucine at the beginning of the TPL (Gould, Sommer, Kroth, et al. 2006; Gruber et al. 2007), but this can be problematic if the signal cleavage site is not confidently predicted. Other confounding factors may well be at play, such as internal cryptic targeting signals, which are known or inferred for up to 15% of plastid targeted proteins in *Arabidopsis* (Zybailov et al. 2008). Nonetheless, we have predicted many cases of dual targeting in *G. theta*, and our experimental localizations support the prediction of dual targeting to the plastid and mitochondrion for tyrRS1 and to the cytosol and PPC for tyrRS2.

Identifying cases of dual targeting is a first step toward a more nuanced understanding of protein targeting in these complex algae. Three main mechanisms can be envisioned to accomplish the dual mitochondrion and plastid targeting identified here. Alternate transcription start sites could produce two distinct transcripts for a given locus, a longer one encoding a plastid BTS, and a shorter one encoding only a mitochondrial presequence. Similarly, alternate translation initiation sites could produce these same two protein isoforms, but from a single transcript. Alternatively, the N-terminal extension may be ambiguous with respect to its binding partners and target the plastid or the mitochondrion depending on whether the signal recognition particle or the mitochondrial import machinery binds it first. Detailed transcription start site mapping and experimental mutation of targeting peptides would allow us to distinguish among these possibilities in diatoms. In cryptophytes, we have additionally predicted dual targeting to the PPC and mitochondrion (glyRS2, Guith1 protein ID 163452, GenBank accession number KP998845), which could be accomplished by any of the same three mechanisms. Dual targeting to the PPC and cytosol, however (tyrRS1, Guith1 protein ID 94450, GenBank accession number KP99887), would be expected to require alternative transcription or translation initiation, because cytosolic localization requires no N-terminal extension. As with diatoms, detailed transcription start site mapping would help us determine the mechanism of dual targeting for each of these proteins. However, experimental localization studies for cryptophytes remain somewhat limited by the lack of a homologous transformation system. Though cryptophyte and diatom plastids are very similar, heterologous localizations artificially impose diatom organelle import requirements on cryptophyte proteins. In both diatoms and cryptophytes, dual or multiple targeting to other combinations of organelles (e.g., mitochondrion and cytosol, PPC and plastid) may still await discovery and would further our understanding of organelle targeting in these highly compartmentalized eukaryotes.

## Supplementary Material

Supplementary tables S1–S4, figure S1 , and file are available at *Genome Biology and Evolution* online (http://www.gbe.oxfordjournals.org/).
